# A Rare Presentation of Invasive Tuberculosis of the Central Nervous System in an Immunocompetent Patient in a Nonendemic Country

**DOI:** 10.1155/2018/2940947

**Published:** 2018-07-09

**Authors:** Rita Martins, Carlos Casimiro, Ana Valverde, Jose Campillo

**Affiliations:** Neurology Department, Hospital Prof. Doutor Fernando Fonseca, Amadora, Portugal

## Abstract

We herein report a rare case of a 25-year-old immunocompetent male patient with disseminated tuberculosis of central nervous system (CNS), first presenting as multiple cerebral lesions with no meningeal involvement. Subsequent diagnostic workup disclosed extensive peritoneal involvement. A broad differential diagnosis was considered, including neoplastic and infectious diseases. The diagnosis was confirmed with positive PCR result for* Mycobacterium tuberculosis* in the biopsied mesenteric tissue. The patient was started on tuberculostatic regimen with favorable outcome. No acquired or hereditary immunodeficiency was documented. Disseminated tuberculosis in immunocompetent individuals is extremely rare. Genetic susceptibility factors have been reported in individuals with extensive forms of the disease and a high index of suspicion is required, as observed in our case.

## 1. Introduction

CNS tuberculosis represents only 5% of extrapulmonary disease manifestations. Neurotuberculosis without pulmonary involvement is extremely rare and disseminated forms in immunocompetent patients are equally very infrequent [1, 2].

## 2. Case Presentation

**Figure 1:**
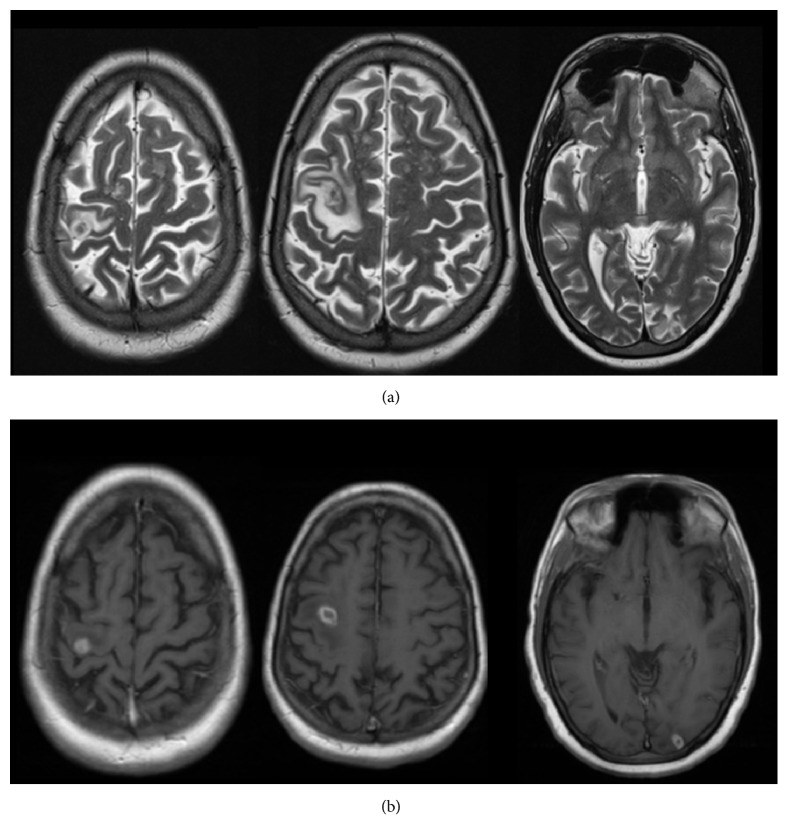
**Brain MRI. (a) Axial T2 weighted: **three nodular lesions, two in right frontal lobe and one in the left occipital lobe, with hypointense central core, hyperintense peripheral margin, and vasogenic oedema.** (b) Axial T1 + gadolinium: **ring-enhancing lesions and absent leptomeningeal enhancement.

A 35-year-old male presented to the emergency department after a tonic-clonic seizure. There was no significant past medical or surgical history. His physical examination was unremarkable, with no fever or focal neurological signs. In the previous 6 months, he reported anorexia and unintentional weight loss of 8 kg, with no other constitutional signs or symptoms. Brain MRI disclosed three ring-enhancing T1 and T2 hypointense cortical lesions, two located in the right frontal lobe and one in the left occipital lobe, associated with vasogenic oedema and absent leptomeningeal enhancement (Figure 1). Based on the imagiological findings, infectious abscesses and metastatic deposits were considered the most probable etiologies. In subsequent diagnostic workup, abdominal CT revealed massive mesenteric infiltration and innumerous lymphadenopathies; therefore neoplastic peritoneal carcinomatosis was first considered. Chest CT and further radiological examination were unremarkable, excluding other organs involved.

**Figure 2:**
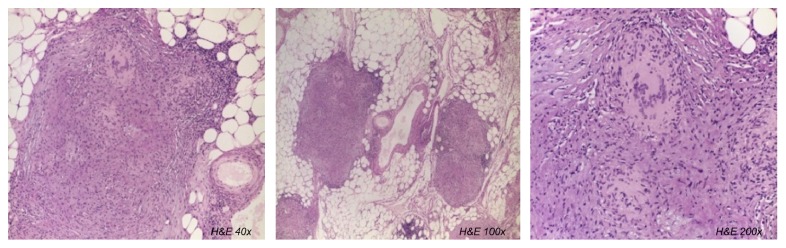
**Histologic examination of mesenteric lesion: **multiple and noncaseating granulomas with a predominantly perivascular distribution (Hematoxylin and Eosin stain, magnification x40, x100, and x200).

Histological examination of the mesenteric lesions revealed multiple noncaseating perivascular granulomas (Figure 2). The polymerase chain reaction (PCR) performed in the tissue specimen was positive for* Mycobacterium tuberculosis*, thus confirming the diagnosis of disseminated tuberculosis of CNS and peritoneum. Extensive laboratory workup for underlying acquired or hereditary immunosuppression was negative, including human immunodeficiency virus testing, immunoglobulin levels, and lymphocyte subset counts. Acid fast bacilli smear, cultures and PCR from sputum, CSF, and blood were negative. The patient was started on tuberculostatic treatment with adjunctive corticosteroids, in a four-drug regimen during the first two months, followed by additional two-drug regimen in the subsequent eight months. He had a favorable outcome, with complete regression of both cerebral and peritoneal lesions.

## 3. Discussion

In nonendemic countries, disseminated tuberculosis is mainly observed in adult immigrants from high prevalence areas and immunocompromised patients [3, 4]. Recent studies have suggested a genetic defect in the interleukin-12 (IL-12) and interferon-gamma pathways as predisposing factors for this disease form. However, the underlying pathophysiology and genetic factors determining the susceptibility to* Mycobacterium tuberculosis* infection in immunocompetent hosts remains unknown. In several animal models, interferon inducible gene signatures have been described to play a crucial role in the host response to mycobacterial infections. Genetically acquired or inherited dysfunction of type I interferons and inflammatory cytokines, such as tumor necrosis factor alpha (TNF-a), IL-12, and IL-1 may reflect the immune propensity to* Mycobacterium tuberculosis* infection. These recent findings represent potential future biomarkers of acute infection [5]; however genetic tests are not yet clinically applied.

Invasive nonmeningeal CNS tuberculosis without evidence of pulmonary involvement is extremely rare and such cases pose an important diagnostic challenge. Neurotuberculosis presents more frequently with tuberculous meningitis, tuberculomas, and spinal arachnoiditis [6, 7]. Intracranial abscesses are an uncommon and may occur with unremarkable CSF analysis [7, 8], as observed in our case.

In conclusion, disseminated nonmeningeal neurotuberculosis with no pulmonary involvement is extremely rare in immunocompetent patients and a high index of suspicion is required in these cases. Genetic susceptibility factors have been recently reported in literature and may predispose individuals to these extensive forms of the disease.
